# Complete mitochondrial genome of *Polyphaga plancyi* (Blattaria: Polyphagidae)

**DOI:** 10.1080/23802359.2016.1214545

**Published:** 2016-09-08

**Authors:** Gang Liu, Mengwei Liu, Wenyu Hui, Chuanqin Chen, Yingyin Zhang

**Affiliations:** a School of Life Science, Anhui Medical University, Hefei, China;; b School of Pharmacy, Anhui Medical University, Hefei, China

**Keywords:** Blattaria, *Polyphaga plancyi*, complete mitochondrial genome sequences

## Abstract

The complete mitochondrial genome of *Polyphaga plancyi* is a 15,547 bp circular molecule, which contains 37 typical mitochondrial genes (13 protein-coding genes, 2 rRNAs and 22 tRNAs) and a 854 bp D-loop. Its gene arrangement pattern is identical with typical other cockroaches. All protein-coding genes start with an ATG codon except COI, ND3, ND5, ND4, ND4L ND6 and ND1. TAA is the most frequent stop codon, and TAG, GCA, TA- and T- are also occurred very common. The mtDNA sequence contains 12S rRNA and 16S rRNA of rRNA. Except for tRNA^Ser(AGY)^ and tRNA^Leu(CUN)^ without the dihydrouridine (DHU) arm, all tRNAs could be folded into canonical cloverleaf secondary structures. The phylogenetic trees from the ML and BI analyses based on the complete mtDNA of nine cockroaches’ species, share similar topologies and high node support values. *Polyphaga plancy* has close relative with *Eupolyphaga sinensis*.

*Polyphaga plancyi* is endemic species belongs to the family Polyphagidae and the order Blattaria, distributed in North China of Hebei and Shanxi Province, and Beijing City. This species is used to be a traditional Chinese medicine, which play an important role in dealing with thrombi (He et al. 2004; Zhang et al. 2010). As an important traditional Chinese medicine, it has been widely artificial feeding in China. Despite a lot of studies about morphological, physiological and biological features were reported, few researches on molecular biology have been done yet.

The samples of *Polyphaga plancyi* was collected from Hejian City, Hebei Province in August, 2015. The samples were stored at medical animals room, in the School of Life Science, Anhui Medical University, P. R. China (Sample codes are AMHU-YXDW20150822). In this study, we determined the complete mtDNA sequence of *Polyphaga plancyi* with the hope of providing more useful molecular information for further studies of traditional Chinese medicine. The complete mtDNA sequence of *Polyphaga plancyi* has been assigned GenBank accession number KU157228.

The length of the complete mtDNA sequence is 15,547 bp, is similar to other Blattaria species (Yamauchi et al. 2004; Xiao et al. 2012). The overall base composition for the mtDNA sequence is as follows: A, 40.9%, C, 17.2%, G, 10.3%, T, 31.6%. A + T content (72.5%) is higher than C + G content (21.5%), similar to other Blattaria species (Zhang et al. 2010; Xiao et al. 2012). The length of intergenic spacer sequences is 62 bp at 11 locations and the overlapping bases are 117 bp existing 19 regions.

Through the 13 PCGs, the longest one is ND5, and the shortest is ATP8, similar with the other species (Zhang et al. 2010; Xiao et al. 2012). All protein-coding genes start with an ATG codon except COI, ND3, ND5, ND4, ND4L ND6 and ND1. TAA is the most frequent stop codon, although ND3, ND4L and ND1 end with TAG, and COI and ATP8 end with TA-, COII end with GCA, and ND4 stop with the single nucleotide T-. The new mtDNA sequence contains a small subunit (12S rRNA) and a large subunit (16S rRNA) of rRNA, which are located between tRNA^Phe^ and D-loop, separated by tRNA^Val^. The 12S rRNA is 711 bp long and the 16S rRNA is 1 295 bp in length. All tRNA genes possess the typical clover leaf secondary structure except for tRNA^Ser(AGN)^ and tRNA^Leu(CUN)^, which lacks a dihydroxyuridine (DHU) arm. The non-coding regions include a control region (D-loop) and a few intergenic spacers. The D-loop is located between 12S RNA and tRNA^Val^, and is 854 bp long.

Phylogenetic trees were estimated using ML and BI methods, based on the complete mtDNA of 9 Blattaria species, and corresponding *Ctenoptilum vasava* (NC_016704) sequence was used as outgroup, sharing similar topologies and high node support values (Figure 1). *Polyphaga plancy* has close relative with *Eupolyphaga sinensis*. The result indicates that these two species have close relationship, which is consistent with the traditional classification (Xiao et al. 2012).

**Figure 1.**
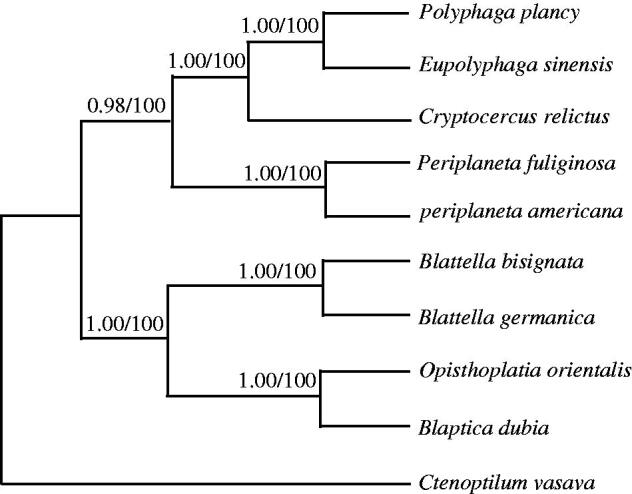
Phylogenetic relationships among the nine cockroaches’ species based on complete mtDNA sequences. Numbers at each node are Bayesian posterior probabilities (left) and maximum likelihood bootstrap proportions (estimated from 100 pseudoreplicates) (right). The accession number in GenBank of 9 Blattaria in this study: *Blattella germanica* (NC_012901), *Blaptica dubia* (KT893459), *Periplaneta fuliginosa* (AB126004), *Opisthoplatia orientalis* (KT893460), *Periplaneta americana* (NC_016956), *Blattella bisignata* (NC_018549), *Cryptocercus relictus* (NC_018132), *Eupolyphaga sinensis* (NC_014274).

## Nucleotide sequence accession number

The complete mtDNA sequence of *Polyphaga plancyi* has been assigned GenBank accession number KU157228.
